# ASSISTED LIVING DIRECT CARE WORKFORCE TRAINING REQUIREMENTS BY LICENSE TYPE

**DOI:** 10.1093/geroni/igad104.1097

**Published:** 2023-12-21

**Authors:** Lindsey Smith, Sarah Dys, Cassandra Hua, Jacy Weems, Kali Thomas

**Affiliations:** Oregon Health & Science University, Portland, Oregon, United States; Portland State University, Portland, Oregon, United States; University of Massachusetts Lowell, Lowell, Massachusetts, United States; Brown University School of Public Health, Providence, Rhode Island, United States; Johns Hopkins University, Baltimore, Maryland, United States

## Abstract

Assisted living (AL) residents and families often assume that care staff have clinical certifications or specialized training. We analyzed direct care worker training requirements for each AL license type across all 50 states and DC, using health services regulatory and content analyses. Across all licenses, we found the most common required training topics specific to care provision were resident rights, providing assistance with daily living, identifying and alleviating safety risks for residents with cognitive impairment, medication management and non-pharmacological interventions, nutrition and hydration, preservation of resident dignity, recognizing and reporting changes in a resident’s condition, understanding resident actions and behavior as communication, and coping with difficult behaviors. We found the most common approach—accounting for the regulations governing 22% of licensed AL communities in the U.S.—was licenses that require between 1 and 10 hours of initial and annual training, but do not have a detailed list of training topics. Only three states (Arizona, Michigan, and New Hampshire) have no training requirements for any unlicensed direct care workers, while 20 states have at least one license without requirements regarding training hours or topics that must be covered. We found that 10 states have a license that requires more than 10 hours of initial and annual training and specifies a detailed list of training requirements. Interestingly, four of these states (New Mexico, Pennsylvania, Virginia, and Washington) have these requirements for all license types, but three of these states (North Carolina, Illinois, and DC) also have a license with no training requirements.

